# Evaluating methods for ranking differentially expressed genes applied to microArray quality control data

**DOI:** 10.1186/1471-2105-12-227

**Published:** 2011-06-06

**Authors:** Koji Kadota, Kentaro Shimizu

**Affiliations:** 1 Agricultural Bioinformatics Research Unit, Graduate School of Agricultural and Life Sciences, The University of Tokyo, 1-1-1 Yayoi, Bunkyo-ku, Tokyo 113-8657, Japan

## Abstract

**Background:**

Statistical methods for ranking differentially expressed genes (DEGs) from gene expression data should be evaluated with regard to high sensitivity, specificity, and reproducibility. In our previous studies, we evaluated eight gene ranking methods applied to only Affymetrix GeneChip data. A more general evaluation that also includes other microarray platforms, such as the Agilent or Illumina systems, is desirable for determining which methods are suitable for each platform and which method has better inter-platform reproducibility.

**Results:**

We compared the eight gene ranking methods using the MicroArray Quality Control (MAQC) datasets produced by five manufacturers: Affymetrix, Applied Biosystems, Agilent, GE Healthcare, and Illumina. The area under the curve (AUC) was used as a measure for both sensitivity and specificity. Although the highest AUC values can vary with the definition of "true" DEGs, the best methods were, in most cases, either the weighted average difference (WAD), rank products (RP), or intensity-based moderated *t *statistic (ibmT). The percentages of overlapping genes (POGs) across different test sites were mainly evaluated as a measure for both intra- and inter-platform reproducibility. The POG values for WAD were the highest overall, irrespective of the choice of microarray platform. The high intra- and inter-platform reproducibility of WAD was also observed at a higher biological function level.

**Conclusion:**

These results for the five microarray platforms were consistent with our previous ones based on 36 real experimental datasets measured using the Affymetrix platform. Thus, recommendations made using the MAQC benchmark data might be universally applicable.

## Background

Identification of differentially expressed genes (DEGs) under different conditions is an important goal in microarray-based gene expression analysis. For this identification, new gene ranking methods have been developed and comparative studies have been performed [1-8]. Several evaluation metrics for comparing these methods have also been used [8-17]. A major metric is the area under the curve (AUC), which evaluates both sensitivity and specificity of the methods simultaneously [8,14-17]. A high AUC value indicates that a set of true DEGs are highly concentrated at the top in ranked gene lists.

Currently, by virtue of the MicroArray Quality Control (MAQC) project [18,19], researchers can comprehensively evaluate many competing methods with regard to reproducibility, another important metric [9-14]. The MAQC study provides a large number of benchmark datasets measured using different microarray platforms and at different test sites for a set of common samples (so-called "Samples A-D"; for details, see Materials and Methods). This enables us to evaluate inter-site (or intra-platform) and inter-platform reproducibility. The study concluded that a fold-change-based method, an average difference (AD) statistic for log-scale data, has the most reproducible results of ranked gene lists when inter-site reproducibility for the DEG lists was evaluated [18]. However, many other existing methods, such as rank products (RP) [4], were not compared in the original study. Moreover, new methods such as weighted average difference (WAD) [8] have been developed since the MAQC study. Evaluations for those methods are therefore important for creating up-to-date guidelines.

We recently reported that WAD outperformed AD, which was recommended by the MAQC study with regard to inter-site reproducibility. We also reported that the use of WAD or RP, in conjunction with suitable preprocessing algorithms dedicated to the Affymetrix (AFX) GeneChip data, can increase both sensitivity and specificity of the results [14]. However, as the datasets we used were derived from only one platform (i.e., the AFX data), the applicability of the conclusions to other platforms, such as Agilent One-Color (AG1) and Applied Biosystems (ABI) data, should be determined.

In this study, we analyzed the MAQC data [18,19] measured using five platforms: ABI, AG1, GE Healthcare (GEH), and Illumina (ILM), as well as AFX. We report the suitable gene ranking methods having high sensitivity, specificity, and inter-site and inter-platform reproducibility for these data. We also report the capability of the recommended methods for generating consistent results at higher biological function levels as well as lower DEG list levels. Our recommendations for the five platforms are essentially the same as the previous ones, which were based on 36 real experimental datasets measured using only the AFX platform [14].

## Methods

### MAQC expression data

Expression data derived from five platforms, i.e., AFX, ABI, AG1, GEH, and ILM, were obtained from the MAQC website [20] and analyzed. The MAQC project produced four sample types: Sample A, a universal human reference RNA; Sample B, a human brain reference RNA; Sample C, consisting of 75 and 25 % Samples A and B respectively; and Sample D, consisting of 25 and 75% Samples A and B respectively [18]. Five replicate experiments for each of the four sample types at three (for ABI, AG1, GEH, and ILM) and six (for AFX) test sites were conducted. The downloaded data were pre-processed using the individual manufacturer protocols. More detail about pre-processing is given in Ref. [18]. The gene ranking methods were independently applied for "Sample A versus B" and "Sample C versus D" comparisons.

For the GEH data, signals from multiple probes with the same ID (e.g., 12 probes with the ID "GE1071362" were present) were averaged. This produced 53,517 unique probes from the original 54,359 probes. Then, signals under a minimum value for signals flagged as 'Good' quality were set to the minimum value (i.e., 0.169). This substitution of a floor value was made for 21.75% (698,424/(53,517 probes × 60 samples)) of the signals. For reproducing the research, the R-code for the preprocessing of GEH data is available in Additional file 1. After the preprocessing of the GEH data, log_2 _transformation was performed. The GEH data, i.e., 53,517 probes, were used for analyzing sensitivity and specificity. For the other platforms (ABI, AFX, AG1, and ILM), the expression data on a log_2 _scale were analyzed.

### MAQC RT-PCR data

Quantitative real-time polymerase chain reaction (RT-PCR) data were obtained from the MAQC website [20] and used for defining "true" DEGs. The original MAQC study [19] produced TaqMan (TAQ) assay data on 997 genes, StaRT-PCR (GEX) assay data on 205 genes, and QuantiGene (QGN) assay data on 244 genes. Of these, we only used the TAQ assay data on 905 genes and the GEX assay data on 161 genes because those genes were common among the five different microarray platforms.

To determine the DEGs, we applied two metrics for the log_2 _transformed data: a *t*-test and an AD statistic (i.e., log-fold-change). The false discovery rate (FDR) for the *p*-values from the *t*-test was calculated using the Benjamini and Hochberg approach [21]. DEGs were selected based on FDR < 0.05 or an absolute AD value > 1. For comparing "Sample A versus B" using the TAQ data, the FDR threshold defined 817 genes as DEGs (*TAQ_AB_FDR *= 817, denoted as "*Platform_Comparison_Metric *") and the AD threshold produced 553 DEGs (*TAQ_AB_AD *= 553). Similarly, 767, 351, 144, 106, 114, and 60 DEGs were determined by *TAQ_CD_FDR, TAQ_CD_AD, GEX_AB_FDR, GEX_AB_AD, GEX_CD_FDR*, and *GEX_CD_AD*, respectively. The R-codes for analyzing the TAQ and GEX data are available in Additional files 2 and 3, respectively.

### Gene ranking

The genes were ranked according to the differential expression using R (ver. 2.11.0) [22] and Bioconductor [23]. Following on from our previous study [8,14], eight methods for analyzing two-class data were compared: the WAD [8], AD [8], fold change (FC), RP [4], moderated *t *statistic (modT) [3], significance analysis of microarrays (samT) [1], shrinkage *t *statistic (shrT) [7], and intensity-based moderated *t *statistic (ibmT) [6]. A higher absolute value for all methods except for RP indicates a higher degree of differential expression. The genes were ranked in descending order of the absolute value.

RP is only a rank-based non-parametric method. Different from the other methods, RP independently handles up-regulated genes and down-regulated genes under one class and therefore produces two separate ranked gene lists. In other words, RP outputs two values per gene. To obtain one RP value per gene for comparison with the results of the other methods, we defined a lower value as a net value for the gene. A small net value for RP is therefore evidence of differential expression. This procedure is the same as that described in Ref. [14]. The genes were ranked in ascending order of the net value.

For evaluation of the methods based both on sensitivity and specificity, an AUC value was calculated for each ranked gene list, where true DEGs were mainly defined by two metrics (i.e., FDR and AD) as mentioned above. For evaluation based on inter-site and inter-platform reproducibility, the percentages of overlapping genes (POGs) and Spearman's rank correlation coefficients between different ranked gene lists were calculated.

### Functional analysis

Analyzing gene set enrichment is a common approach in functional analysis where functionally or structurally related sets of genes regarding differential expression are assessed. Methods for this purpose include Gene Set Enrichment Analysis (GSEA) [24] and Parametric Analysis of Gene Set Enrichment (PAGE) [25]. These methods calculate the differences between two classes of samples (e.g., Sample A versus B) by using associative statistics, such as a signal-to-noise metric [24] or AD [25], and output one score per gene set. Different associative statistics with a particular method (e.g., GSEA with AD and GSEA with samT) could result in different top-rank lists of gene sets.

To compare the performances of the eight gene ranking methods as simply as possible, we calculate the average of the ranks of the genes belonging to a gene set as an "enrichment score". Consider a gene ranking method that produces *m *associative statistics ***S ***= {*s_1_, s_2_*, ..., *s_m_*} composed of *m *genes. Given ***R ***= {*r_1_, r_2_*, ..., *r_m_*} as the corresponding ranks of the values of the statistics and ***G ***= {*g_1_, g *_2_, ..., *g_n_*} as a gene set composed of *n *genes, the enrichment score for the gene set, *E_G_*, is simply calculated as. For example, consider three genes in a gene set ranked as 2^nd^, 8^th^, and 14^th ^respectively. The enrichment score is simply calculated as *E_G_*= (2+8+14)/3 = 8. Accordingly, if a gene set has a small *E_G_*value, it is expected to be enriched (or de-regulated) in the two classes analyzed.

The gene sets were obtained from the Molecular Signatures Databases (MSigDB) ver. 3.0 [24]. We used 186 gene sets associated with the Kyoto Encyclopedia of Genes and Genomes (KEGG) Pathway. Since the analysis of gene set enrichment provides an enrichment score for each gene set, we obtained 186 enrichment scores for each gene ranking method. The pathways were analyzed for each of the eight gene ranking methods, each of the two comparisons ("Sample A versus B" and "C versus D"), each of the three test sites, and each of the five microarray platforms. Accordingly, the analysis produced a total of (8 × 2 × 3 × 5 =) 240 lists, each of which had 186 scores. Since the comparisons were performed for each platform, each list was denoted as "*Method_Comparison_Site *".

Gene set information is available as gene symbols, while expression data are measured on the probe basis. Inevitably, many multiple probes are mapped to the same gene on existing microarray platforms. For example, three probes ('201127_s_at', '201128_s_at', and '210337_s_at') are mapped to the ACLY gene on the AFX platform, while only one probe is mapped to that gene on the other platforms ('102218' for ABI, '17711' for AG1, '199102' for GEH, and 'GI_38569420-S' for ILM). In this case, expression signals for the three probes on the AFX platform should be collapsed to avoid inflating the enrichment scores and to facilitate biological interpretation of the analysis results [24]. We averaged the signals from the probes mapped to the same gene.

The mapping information for the five platforms was obtained from the Gene Expression Omnibus [26] as 'GPL570' for AFX, 'GPL2986' for ABI, 'GPL1708' for AG1, 'GPL2895' for GEH, and 'GPL2507' for ILM. For the GEH data, the original data composed of 54,359 probes were collapsed. After this, expression data composed of 20,723 unique genes for AFX, 16,275 for ABI, 19,053 for AG1, 19,352 for GEH, and 16,992 for ILM on a log_2 _scale were analyzed. The POGs and Spearman's rank correlation coefficients for the 186 enrichment scores between test sites (or different platforms) were calculated for estimating inter-site (or inter-platform) reproducibility of the individual gene ranking methods at the function level.

## Results

Investigation of suitable gene ranking methods having high sensitivity, specificity, and (inter-site and inter-platform) reproducibility is the main purpose of this work. Similar to our previous study on the AFX platform [14], eight gene ranking methods (WAD, AD, FC, RP, modT, samT, shrT, and ibmT) were evaluated. Compared to the abundant real experimental data measured using the AFX platform in our previous study, the data for other platforms are considerably limited. We therefore analyzed the MAQC data measured using five platforms (AFX, ABI, AG1, GEH, and ILM). The results for the MAQC AFX data were compared with our previous results, where 36 real experimental datasets measured using the AFX platform were analyzed [14]. We will follow the terminology used in our previous study [14] for consistency.

### Sensitivity and specificity

We and other researchers believe the human samples compared in the MAQC study, cell lines (sample A) versus brain tissues (sample B), are clearly distinct, i.e., such comparisons are unrealistic in practical biological research [27-29]. This may weaken the confidence of any results obtained using the MAQC benchmark data. To mitigate this concern, we first analyzed the MAQC data measured using the AFX platform and compared the current results to our previous ones (where the best method was either WAD or RP) [14]. The evaluation frameworks were set to be as similar as possible to those in the previous study [14]. Two assay technologies for RT-PCR data (TAQ and GEX) [19] were used to determine true DEGs, and an AUC value was used to compare sensitivity and specificity simultaneously [30-32]. Currently, there is no convincing rationale for defining DEGs from the RT-PCR data. We here define DEGs by two metrics: a FDR threshold (FDR < 0.05) and an absolute AD threshold (|AD| > 1; AD corresponds to "log-fold-change").

The average AUC values for the AFX data across six test sites are shown in Table 1. WAD and RP, recommended by Ref. [14], show the best performance in six of the eight cases. Note that the ibmT method also shows high AUC values as a whole; it is always the second best when WAD is the best. These observations are similar to those in our previous study [14], suggesting that findings derived from the MAQC data measured using the other four platforms (ABI, AG1, GEH, and ILM) could be applicable for practical microarray research.

**Table 1:** Average AUC values for AFX data

Technology	TAQ	TAQ	TAQ	TAQ	GEX	GEX	GEX	GEX
Comparison	AB	AB	CD	CD	AB	AB	CD	CD
Metric	FDR	AD	FDR	AD	FDR	AD	FDR	AD
#DEGs	817	553	767	351	144	106	114	60
WAD	**69.91**	75.74	**71.41**	77.69	**75.74**	**78.75**	**76.54**	80.69
AD	67.73	77.45	69.19	81.24	70.77	76.60	71.26	78.96
FC	67.73	77.44	69.17	81.20	70.79	76.61	71.35	78.95
RP	67.37	77.43	68.85	**81.78**	70.61	76.36	71.19	79.46
modT	68.74	76.98	70.19	79.95	73.12	77.67	73.53	80.77
samT	67.96	**77.52**	69.49	81.05	71.26	76.87	71.93	79.61
shrT	68.64	77.11	70.16	80.08	72.90	77.64	73.39	80.68
ibmT	69.26	76.85	70.70	79.57	74.27	78.26	74.87	**81.33**

Average AUC values across six test sites. Highest AUC values for each case are in bold. A total of 8 cases (2 assay technologies × 2 comparisons × 2 metrics) are shown. For example, AUC value for samT (77.52) was the highest in *TAQ_AB_AD *case, where 553 genes were defined as DEGs.

The average AUC values for ABI, AG1, GEH, and ILM data across three test sites are shown in Table 2. Similar to the results for the AFX data, the best was either WAD, RP, or ibmT in all but one (i.e., *TAQ_AB_AD *) case. The ibmT method may be suitable for ABI data. RP was overall good when DEGs were determined by the AD metric for TAQ RT-PCR data. WAD was overall good when DEGs were determined by the FDR metric from a *t*-test, regardless of the choice of RT-PCR technology (i.e., TAQ or GEX).

**Table 2:** Average AUC values for ABI, AG1, GEH, and ILM data

Technology	TAQ	TAQ	TAQ	TAQ	GEX	GEX	GEX	GEX
Comparison	AB	AB	CD	CD	AB	AB	CD	CD
Metric	FDR	AD	FDR	AD	FDR	AD	FDR	AD
#DEGs	817	553	767	351	144	106	114	60
*ABI *								
WAD	66.18	74.77	67.30	77.46	70.71	74.49	70.01	76.62
AD	63.45	75.49	62.88	78.92	65.33	71.14	61.86	71.10
FC	62.93	75.00	62.27	78.63	64.95	70.76	60.84	70.42
RP	62.46	75.45	60.28	**79.58**	64.17	70.43	58.37	68.82
modT	66.03	73.63	67.62	76.75	71.27	74.84	70.54	77.31
samT	64.00	**75.63**	63.80	79.23	66.36	71.92	63.34	72.57
shrT	65.60	74.91	66.86	78.05	69.75	74.12	68.80	76.54
ibmT	**66.37**	73.43	**67.93**	76.41	**71.99**	**75.26**	**71.12**	**77.39**
*AG1 *								
WAD	**66.73**	73.14	**67.39**	74.69	**71.28**	75.58	**72.67**	77.80
AD	64.60	75.20	64.41	78.48	65.73	73.53	65.75	75.65
FC	64.32	75.15	63.01	77.55	65.42	73.58	63.98	73.95
RP	64.37	**75.20**	65.03	**79.35**	65.38	72.78	66.45	76.02
modT	65.66	74.25	66.00	76.42	68.80	74.78	70.44	77.75
samT	64.26	74.29	64.77	78.66	66.34	73.79	66.49	76.24
shrT	65.47	74.78	65.67	77.76	68.01	74.65	69.19	77.89
ibmT	65.97	74.32	66.34	76.59	69.80	**75.61**	71.22	**78.86**
*GEH *								
WAD	**74.03**	78.39	**74.64**	79.38	**76.18**	**79.19**	**76.87**	**81.26**
AD	71.27	78.60	71.24	80.36	70.50	75.64	70.09	77.28
FC	71.16	78.50	70.81	80.21	70.30	75.48	69.84	77.05
RP	71.19	**78.68**	70.97	**80.62**	70.58	75.71	69.93	77.54
modT	71.90	78.04	71.74	79.01	71.18	75.46	72.25	78.26
samT	71.40	78.66	71.54	80.48	70.61	75.69	70.72	77.76
shrT	72.02	78.64	72.12	80.13	71.30	75.89	72.19	78.63
ibmT	71.87	78.01	71.70	78.99	71.14	75.41	72.27	78.31
*ILM *								
WAD	**79.13**	82.49	**78.94**	82.63	**83.25**	**84.79**	**82.09**	83.88
AD	77.43	83.85	76.33	84.64	79.92	83.71	77.93	82.93
FC	77.39	83.83	76.35	84.63	79.90	83.77	78.14	83.17
RP	77.58	**84.23**	76.10	**85.28**	79.81	83.94	77.97	**84.13**
modT	77.45	82.98	76.73	83.56	80.64	83.78	78.70	82.96
samT	77.42	83.63	76.53	84.39	80.08	83.70	78.28	83.02
shrT	77.39	83.05	76.72	83.58	80.49	83.73	78.64	82.93
ibmT	77.94	82.96	77.53	83.48	81.37	84.04	79.77	83.31

Average AUC values across three test sites. Reminder of legend is the same as in Table 1.

Note that these results were based on only two sets of DEGs arbitrarily defined by commonly used procedures (i.e., FDR < 0.05 and |AD| > 1). As the AUC values vary with the thresholds for individual metrics, the effects of changing the parameters should also be analyzed. Table 3 shows the gene ranking methods having the highest AUC values when the top *X *genes are defined as DEGs (*X *= 100, 200, 300, ..., and 800 for TAQ data and 50, 100, and 150 for GEX data). We confirmed the superiority of the WAD, RP, and ibmT methods against various thresholds.

**Table 3:** List of best methods with different threshold settings for defining DEGs

Technology	TAQ	TAQ	TAQ	TAQ	TAQ	TAQ	TAQ	TAQ	GEX	GEX	GEX
#DEGs	100	200	300	400	500	600	700	800	50	100	150
*AFX *											
AB_FDR	WAD	WAD	WAD	WAD	WAD	WAD	WAD	WAD	WAD	WAD	WAD
AB_AD	RP	AD	AD	RP	RP	samT	ibmT	WAD	RP	WAD	WAD
CD_FDR	AD	RP	WAD	WAD	WAD	WAD	WAD	WAD	WAD	WAD	WAD
CD_AD	AD	RP	RP	RP	RP	RP	ibmT	WAD	ibmT	WAD	WAD
*ABI *											
AB_FDR	WAD	ibmT	WAD	WAD	WAD	WAD	WAD	ibmT	ibmT	ibmT	ibmT
AB_AD	RP	RP	RP	RP	RP	samT	WAD	ibmT	samT	ibmT	ibmT
CD_FDR	WAD	WAD	WAD	WAD	ibmT	ibmT	ibmT	ibmT	WAD	ibmT	ibmT
CD_AD	RP	RP	RP	samT	shrT	WAD	ibmT	ibmT	ibmT	ibmT	ibmT
*AG1 *											
AB_FDR	RP	WAD	WAD	ibmT	WAD	WAD	WAD	WAD	WAD	WAD	WAD
AB_AD	RP	RP	RP	RP	RP	RP	ibmT	WAD	FC	ibmT	WAD
CD_FDR	shrT	WAD	WAD	WAD	WAD	WAD	WAD	WAD	ibmT	WAD	WAD
CD_AD	FC	RP	RP	RP	RP	RP	WAD	WAD	ibmT	ibmT	WAD
*GEH *											
AB_FDR	RP	WAD	WAD	WAD	WAD	WAD	WAD	WAD	WAD	WAD	WAD
AB_AD	RP	RP	RP	RP	RP	shrT	WAD	WAD	WAD	WAD	WAD
CD_FDR	WAD	WAD	WAD	WAD	WAD	WAD	WAD	WAD	WAD	WAD	WAD
CD_AD	samT	RP	RP	RP	RP	WAD	WAD	WAD	WAD	WAD	WAD
*ILM *											
AB_FDR	AD	WAD	WAD	WAD	WAD	WAD	WAD	WAD	WAD	WAD	WAD
AB_AD	RP	RP	RP	RP	RP	RP	RP	WAD	RP	WAD	WAD
CD_FDR	RP	RP	WAD	WAD	WAD	WAD	WAD	WAD	WAD	WAD	WAD
CD_AD	AD	RP	RP	RP	RP	RP	WAD	WAD	RP	WAD	WAD

For reproducing the research, the R-codes for obtaining the AUC values shown in Tables 1 and 2 are given in Additional files 4, 5, 6, 7 and 8. The raw AUC values for all methods shown in Table 3 and the R-code for obtaining a fraction of the values are given in Additional files 9 and 10, respectively.

### Inter-site reproducibility at lower DEG list level

Next, we examined the inter-site reproducibility of ranked gene lists among three test sites for each platform. Our previous study using AFX data reports that WAD is the most reproducible method when POG is evaluated (Figure one in Ref. [14]). The POG values for the 100 top-ranked genes among three test sites for the five platforms AFX, ABI, AG1, GEH, and ILM are shown in Table 4. The complete POG values are given in Additional files 11 and 12.

**Table 4:** Inter-site reproducibility at lower DEG list level

Comparison	AB	AB	AB	AB	AB	CD	CD	CD	CD	CD
Platform	AFX	ABI	AG1	GEH	ILM	AFX	ABI	AG1	GEH	ILM
WAD	**88**	**86**	**85**	**81**	83	**64**	**76**	**54**	**39**	**66**
AD	76	76	73	**81**	**85**	23	11	2	5	18
FC	77	72	59	78	84	23	0	0	3	19
RP	78	72	76	79	78	20	8	2	7	9
modT	16	26	30	8	27	20	26	3	1	13
samT	71	63	42	73	45	32	31	5	10	21
shrT	17	33	43	23	28	21	40	2	1	12
ibmT	21	32	37	6	28	29	39	10	1	22

POG values for 100 top-ranked genes among three test sites. For AFX data, POG values were calculated based on first three test sites.

The POG values for WAD were clearly higher than those for the other seven methods, especially in the "Sample C versus D" comparison. The results of that comparison should take precedence over those of "Sample A versus B". This is because sample C is a 3:1 and sample D is a 1:3 mixture of samples A and B, respectively, and real biological samples consist of various kinds of tissues and cells. These results suggest that the use of WAD can be promising for users concerned about inter-site reproducibility at the DEG list level.

**Figure 1:**
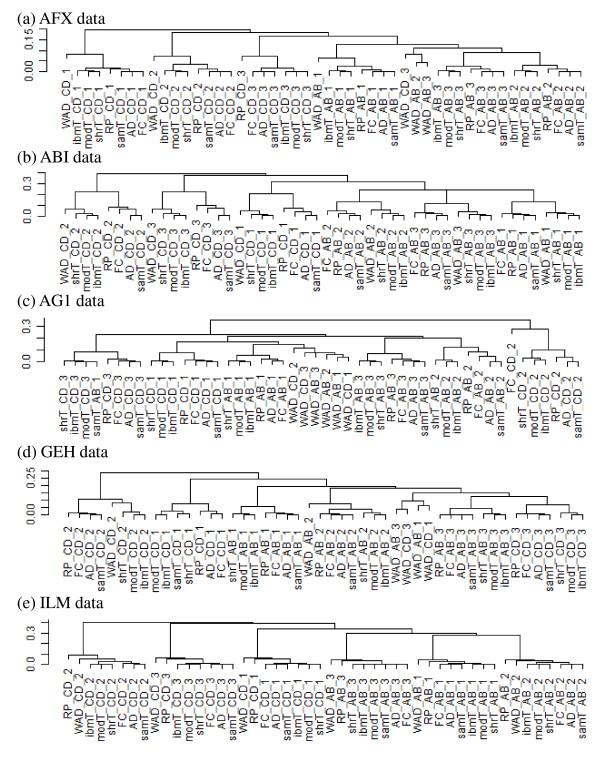
**Dendrograms of average-linkage hierarchical clustering**. Dendrograms for (a) AFX, (b) ABI, (c) AG1, (d) GEH, and (e) ILM data. Spearman's rank correlation coefficient is used as a similarity metric; left-hand scale represents (1- correlation coefficient). Each gene list is denoted as "*Method_Comparison_Site *". For example, a ranked list calculated from samT when comparing C versus D for site 2 data is denoted as "samT_CD_2".

Note that the POG values shown in Table 4 only describe inter-site (or intra-platform) reproducibility among three test sites for each gene ranking method. Accordingly, there may be concern about the potential difference of the ranked gene lists between WAD and the other methods. Figure 1 shows the results of hierarchical clustering applied to a total of 48 ranked gene lists (8 methods × 2 comparisons × 3 test sites = 48 lists; each list is therefore denoted as "*Method_Comparison_Site *"). Each list consists of (a) 54,676 gene ranks for AFX data, (b) 32,879 for ABI, (c) 41,059 for AG1, (d) 53,517 for GEH, and (e) 47,294 for ILM. When we focus on the dendrograms for the ILM data (Figure 1e), six distinct clusters can be seen, each consisting of eight lists with the same sample comparison and the same test site but different gene ranking methods. We also observe this trend for the ABI data (Figure 1b). Different from those for the ILM and ABI data, the dendrogram for the AG1 data shows a distinct WAD cluster (Figure 1c). This cluster is clearly located in a larger cluster mainly consisting of gene lists from the "Sample A versus B" comparison. These results indicate that the ranked gene lists produced from WAD are undoubtedly not outliers.

Similar to the POG values in Table 4 the inter-site reproducibility can also be evaluated by calculating the Spearman's rank correlation coefficients of two ranked gene lists. The high inter-site reproducibility of WAD was also confirmed using this metric. The correlation coefficients between any two test sites (i.e., "Site 1 versus 2", "1 versus 3", and "2 versus 3") are given in Additional file 13.

### Inter-site reproducibility at higher biological function level

In general, the higher the reproducibility of a gene ranking method at a lower DEG list level, the higher the reproducibility the method should have at a higher biological function level. To clarify this idea, we performed a functional analysis by estimating gene set enrichment. There are many sophisticated methods for analyzing gene set enrichment, such as GSEA [24] and PAGE [25]. We calculated the average rank for genes belonging to each gene set as the "enrichment score." This was to manipulate the eight gene ranking methods equally and to perform the comparison as simply as possible (for details, see Materials and Methods).

**Figure 2:**
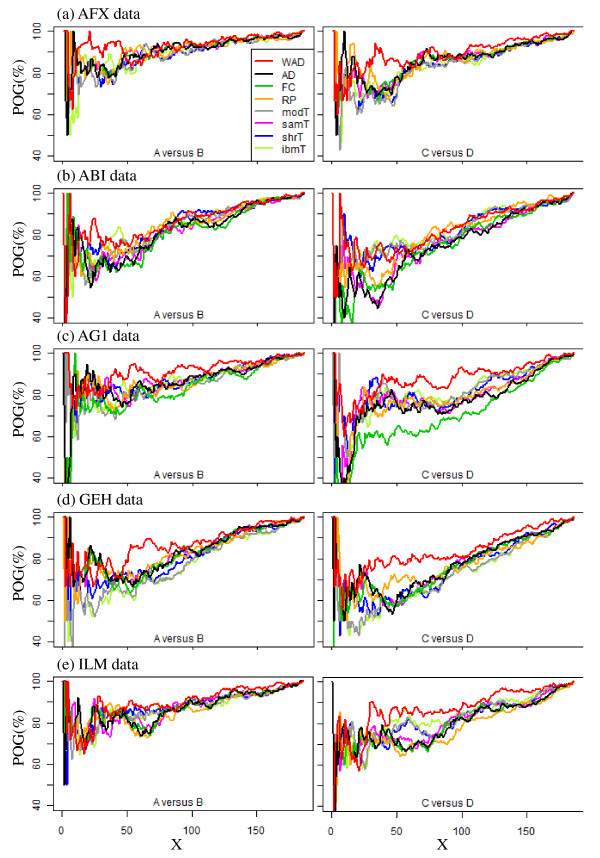
**Inter-site reproducibility at higher biological function level**. POG values for different settings of *X *top-ranked gene sets among three test sites. For AFX data, POG values were calculated based on first three test sites.

We performed the functional analysis for 186 gene sets associated with KEGG Pathway. The POG values for different settings of *X *top-ranked gene sets among three test sites for the five platforms (AFX, ABI, AG1, GEH, and ILM) are shown in Figure 2. While the best methods depend on the number of top-ranked gene sets, WAD seems to have relatively good performance compared to the other methods. We also observed this trend when Spearman's rank correlation coefficients were evaluated as another metric (see Additional file 14). The raw data for Figure 2 are available in Additional file 15. The R-code for making the left side of Figure 2(a) is also available in Additional file 16.

### Inter-platform reproducibility at higher biological function level

Note that the numbers of genes belonging to each gene set differ across platforms. Only 5 of the 186 KEGG Pathway gene sets (*STEROID_BIOSYNTHESIS, VALINE_LEUCINE_AND_ISOLEUCINE_BIOSYNTHESIS, FOLATE_BIOSYNTHESIS, TERPENOID_BACKBONE_BIOSYNTHESIS*, and *RENIN_ANGIOTENSIN_SYSTEM *) were identical across the 5 platforms. To estimate the overall similarity among platforms, we calculated an *intersection-union ratio *for each gene set. For example, there are 61, 61, 60, 54, and 61 genes in the respective platforms AFX, ABI, AG1, GEH, and ILM in the *GLYCOLYSIS_GLUCONEOGENESIS *pathway. As the intersection and union across the 5 platforms are 53 and 61, respectively, the ratio is 53/61 = 0.87. Detailed information on the 186 gene sets is available in Additional file 17. The average of the 186 intersection-union ratios was 0.83. Arguably, we assert this high value enables a comparison between platforms.

**Figure 3:**
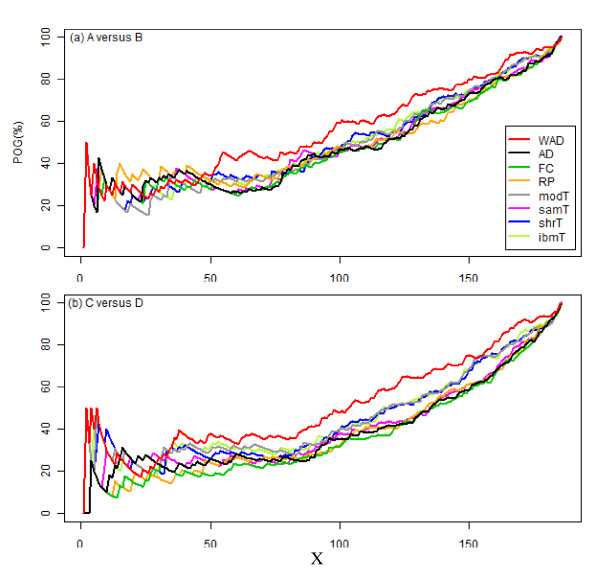
**Inter-platform reproducibility at higher biological function level**. POG values for different settings of *X *top-ranked gene sets among five platforms. Values correspond to intersections among five platforms shown in Figure 2 when comparing (a) A versus B and (b) C versus D.

The POG values for different settings of *X *top-ranked gene sets among the five platforms are shown in Figure 3. Similar to the results for inter-site reproducibility shown in Figure 2, the results for inter-platform reproducibility do not indicate the best gene ranking method because one method does not always outperform the others, especially when comparing ~25% of the top-ranked gene sets. This result is somewhat disappointing because only ~20 top-ranked gene sets have been evaluated at most [13,33]. Nevertheless, as a whole, WAD outperforms the others. We also observed this trend for the 825 Gene Ontology gene sets (file name "c5.bp.v3.0.symbols.gmt") and for all 3,272 curated gene sets ("c2.all.v3.0.symbols.gmt"), obtained from MSigDB ver. 3.0 [24] (data not shown). The raw data for Figure 3 are available in Additional file 18.

## Discussion

We investigated suitable gene ranking methods across various microarray platforms using a large set of MAQC microarray data. In accordance with previous evaluation frameworks [8,14], sensitivity and specificity of the methods were evaluated using an AUC metric where genes were determined to be DEGs or non-DEGs based on RT-PCR data. Reproducibility was evaluated using a POG metric and Spearman's correlation coefficient. The current conclusions are essentially the same as those of our previous studies [8,14]: the best of the eight methods investigated was (i) either WAD, RP, or ibmT, when both sensitivity and specificity were evaluated and (ii) WAD when reproducibility was evaluated. As we proposed one of the methods (WAD) that is compared here, this study could be regarded as the third in a series of studies on WAD. In the first study [8], the high sensitivity and specificity of the above-mentioned methods (especially WAD) were demonstrated using two spike-in datasets [34,35] and 36 real experimental datasets. In the second study [14], the sensitivity and specificity of the eight gene ranking methods coupled with nine pre-processing algorithms dedicated to the Affymetrix (AFX) GeneChip data were further evaluated using the 36 real experimental datasets, and high reproducibility of WAD was demonstrated using the MAQC data. However, our previous studies were based only on datasets measured using the AFX platform. We have demonstrated in this study that those recommendations are also applicable for the other four microarray platforms.

### Sensitivity and specificity

The current results indicate that care should be taken when choosing both a gene ranking method for analyzing microarray data and a metric for analyzing RT-PCR data. In addition, there are more effective combinations of the two choices: the WAD method in conjunction with the FDR metric (WAD/FDR), RP/AD, and ibmT/FDR. These combinations are logical because RP/AD are both fold-change-based statistics and ibmT/FDR are both *t*-statistic-based ones. Although WAD is a fold-change-based method, the overall performance of WAD/FDR is superior to that of ibmT/FDR (Table 3). To our knowledge, no study has reported suitable combinations of the two choices to facilitate agreement between the results. However, note that excessive value may have been placed on the RT-PCR data when determining these combinations. Although the RT-PCR technology is generally considered the "gold-standard" assay for measuring gene expression by biologists [36], it has not been proven as the best. In this regard, our proposed combinations may be useful for discussion on the agreement or disagreement between the results obtained by the two technologies.

### Reproducibility

As mentioned, our previous study reports that WAD outperforms the other methods when inter-site reproducibility is evaluated for AFX microarray data [14]. In the current study, we demonstrate that WAD has clearly higher inter-site reproducibility when DEG lists produced from different test sites are compared, irrespective of the choice of microarray platform (Table 4 and Additional files 11, 12).

Li et al. recently reported high inter-platform reproducibility at the biological function level obtained by using a commercially available tool [13]. Only the samT method [1] was used in their functional analysis, without any justification, despite citation of the original MAQC paper where AD was recommended [18]. In addition, there should be "levels" of reproducibility; the use of better gene ranking methods should increase the level of inter-platform reproducibility at the biological function level. However, comparison of many gene ranking methods at the biological function level using such specialized tools is difficult practically for the following reasons: (1) gene ranking methods of interest may not be used, (2) only a set of significant gene sets can be manipulated, and so on. In particular, related to reason (1), to our knowledge there is no method that can also analyze gene set enrichment in conjunction with non-parametric gene ranking methods such as RP [4]. In this sense, our simple non-parametric approach to estimating gene set enrichment can be deemed a new method. Our functional analysis using this simple approach demonstrates that WAD has both high intra- and inter-platform reproducibility at the biological function level (Figures 2 and 3). We observed similarity between the top-rank lists produced by this simple procedure and those by two widely used methods (i.e., GSEA [24] and PAGE [25]) when some real experimental datasets used in Refs. [24,25,37] were applied (data not shown), but such a comparison is out of the scope of this study. Improving the approach of using WAD for functional analysis is future work.

In this study, we could perform a more detailed analysis with regard to reproducibility by using MAQC datasets. However, the original MAQC study [18] has often been criticized for its evaluation framework, where the correlation coefficient and the POG were used as measures for reproducible ranked gene lists [27-29]. Chen et al. [29] stated that the POG metric does not reflect the accuracy (i.e., sensitivity and specificity) of a ranked gene list. This is true in a sense because, for example, POG values higher than those of WAD can easily be obtained if we rank genes according to relative average signal intensity (i.e., a weight term (*w *) in a WAD statistic; see Figure one in Ref. [14]). This *w *statistic is clearly inferior to that obtained from the other specialized gene ranking methods such as WAD when the AUC value (both sensitivity and specificity) is evaluated [8,14]. Evaluation based only on the reproducibility of ranked gene lists is therefore insufficient. In the current study, however, we compare the eight gene ranking methods using the AUC value (both sensitivity and specificity) as well as the POG value (reproducibility). In addition, the most reproducible method (i.e., WAD) is one of the three methods that found to have high sensitivity and specificity.

### Other pre-processing methods

In this study, we analyzed publicly available datasets that were already pre-processed according to the MAQC philosophy [18]. Our additional processing for the datasets was basically only the log_2 _transformation (see Materials and Methods). As demonstrated by our previous study [14] and another study of the MAQC project [38], there are many other choices for pre-processing data (for example, see Refs. [39-42]) and different choices may result in different conclusions. Thus, the other pre-processing choices should be evaluated next.

## Conclusion

In conclusion, the superiority of WAD was confirmed with regard to sensitivity, specificity, and reproducibility by using the MAQC datasets. We thus recommend the use of WAD for more effective transcriptome analysis.

## List of abbreviations used

ABI: Applied Biosystems; AD: average difference; AFX: Affymetrix; AG1: Agilent one-color microarray; AUC: area under the curve; FDR: false discovery rate; GEH: GE Healthcare; GEX: StaRT-PCR; ibmT: intensity-based moderated *t*-statistic; ILM: Illumina; MAQC: MicroArray Quality Control (project); POG: percentage of overlapping genes; RP: rank products; samT: significance analysis of microarrays; shrT: shrinkage *t*-statistic; TAQ: TaqMan; WAD: weighted average difference (method)

## Authors' contributions

KK performed the analyses and wrote the paper. KS supervised the critical discussion by KK. All authors read and approved the final manuscript.

## Supplementary Material

Additional file 1 **R-code for pre-processing GEH data**. Input file must be obtained from http://edkb.fda.gov/MAQC/MainStudy/upload/norm_MAQC_GEH_123_Median1.zip. After execution of this R-code, expression data consisting of 53,517 unique probes can be obtained as an output file arbitrarily named "norm_MAQC_GEH_123_Median1_unique.txt". Note that the website with the normalized MAQC data was unavailable under review. The raw data may be availabl from. http://www.fda.gov/ScienceResearch/BioinformaticsTools/Arraytrack/ or http://www.ncbi.nlm.nih.gov/geo/query/acc.cgi?acc=GSE5350. Click here for file

Additional file 2 **R-code for analyzing TAQ data**. Two input files must first be obtained from the following websites: "http://www.nature.com/nbt/journal/v24/n9/extref/nbt1239-S5.txt" and "http://edkb.fda.gov/MAQC/MainStudy/upload/norm_TAQ_1_POLR2A.zip". After execution of this R-code, FDR and AD statistics for each comparison (i.e., "Sample A versus B" and "C versus D") can be obtained in an output file arbitrarily named "data_TAQ.txt". Note that the website with the normalized MAQC data was unavailable under review. The raw data may be available from http://www.fda.gov/ScienceResearch/BioinformaticsTools/Arraytrack/ or http://www.ncbi.nlm.nih.gov/geo/query/acc.cgi?acc=GSE5350. Click here for file

Additional file 3 **R-code for analyzing GEX data**. Two input files must first be obtained from the following websites: "http://www.nature.com/nbt/journal/v24/n9/extref/nbt1239-S5.txt" and "http://edkb.fda.gov/MAQC/MainStudy/upload/norm_MAQC_GEX_1_ACTB.zip". After execution of this R-code, FDR and AD statistics for each comparison (i.e., "Sample A versus B" and "C versus D") can be obtained in an output file arbitrarily named "data_GEX.txt". Note that the website with the normalized MAQC data was unavailable under review. The raw data may be available from http://www.fda.gov/ScienceResearch/BioinformaticsTools/Arraytrack/ or http://www.ncbi.nlm.nih.gov/geo/query/acc.cgi?acc=GSE5350. Click here for file

Additional file 4 **R-code for calculating average AUC values for AFX data across six test sites shown in Table 1**. Two input files are required. One is the AFX gene expression data file obtained from http://edkb.fda.gov/MAQC/MainStudy/upload/norm_MAQC_AFX_123456_qPLIER16.zip. The other includes the DEG information derived from individual RT-PCR data. Specifically, the filename should be "data_TAQ.txt" (default) or "data_GEX.txt", generated by executing Additional file 2 or 3, respectively. After execution of this R-code, the average AUC values shown in Table 1 can be obtained in an output file arbitrarily named "result_AFX.txt". Note that the website with the normalized MAQC data was unavailable under review. The raw data may be available from http://www.fda.gov/ScienceResearch/BioinformaticsTools/Arraytrack/ or http://www.ncbi.nlm.nih.gov/geo/query/acc.cgi?acc=GSE5350. Click here for file

Additional file 5 **R-code for calculating average AUC values for ABI data across three test sites shown in Table 2**. Two input files are required. One is the ABI gene expression data file obtained from http://edkb.fda.gov/MAQC/MainStudy/upload/norm_MAQC_ABI_123_qNorm.zip. The other includes the DEG information derived from individual RT-PCR data. Specifically, the filename should be "data_TAQ.txt" (default) or "data_GEX.txt", generated by executing Additional file 2 or 3, respectively. After execution of this R-code, the average AUC values for ABI data shown in Table 2 can be obtained in an output file arbitrarily named "result_ABI.txt". Note that the website with the normalized MAQC data was unavailable under review. The raw data may be available from http://www.fda.gov/ScienceResearch/BioinformaticsTools/Arraytrack/ or http://www.ncbi.nlm.nih.gov/geo/query/acc.cgi?acc=GSE5350. Click here for file

Additional file 6 **R-code for calculating average AUC values for AG1 data across three test sites shown in Table 2**. Two input files are required. One is the AG1 gene expression data file obtained from http://edkb.fda.gov/MAQC/MainStudy/upload/norm_MAQC_AG1_123_Median1GeneSpring.zip. The other includes the DEG information derived from individual RT-PCR data. Specifically, the filename should be "data_TAQ.txt" (default) or "data_GEX.txt", generated by executing Additional file 2 or 3, respectively. After execution of this R-code, the average AUC values for AG1 data shown in Table 2 can be obtained in an output file arbitrarily named "result_AG1.txt". Note that the website with the normalized MAQC data was unavailable under review. The raw data may be available from http://www.fda.gov/ScienceResearch/BioinformaticsTools/Arraytrack/ or http://www.ncbi.nlm.nih.gov/geo/query/acc.cgi?acc=GSE5350. Click here for file

Additional file 7 **R-code for calculating average AUC values for GEH data across three test sites shown in Table 2**. Two input files are required. One is the GEH gene expression data file obtained by executing Additional file 1, and the output file should be named "norm_MAQC_GEH_123_Median1_unique.txt". The other includes the DEGs information derived from individual RT-PCR data. Specifically, the filename should be "data_TAQ.txt" (default) or "data_GEX.txt", generated by executing Additional file 2 or 3, respectively. After execution of this R-code, the average AUC values for GEH data shown in Table 2 can be obtained in an output file arbitrarily named "result_GEH.txt". Click here for file

Additional file 8 **R-code for calculating average AUC values for ILM data across three test sites shown in Table 2**. Two input files are required. One is the ILM gene expression data file obtained from http://edkb.fda.gov/MAQC/MainStudy/upload/norm_MAQC_ILM_123_qNorm16.zip. The other includes the information about DEGs derived from individual RT-PCR data. Specifically, the filename should be "data_TAQ.txt" (default) or "data_GEX.txt", generated by executing Additional file 2 or 3, respectively. After execution of this R-code, the average AUC values for ILM data shown in Table 2 can be obtained in an output file arbitrarily named "result_ILM.txt". Note that the website with the normalized MAQC data was unavailable under review. The raw data may be available from http://www.fda.gov/ScienceResearch/BioinformaticsTools/Arraytrack/ or http://www.ncbi.nlm.nih.gov/geo/query/acc.cgi?acc=GSE5350. Click here for file

Additional file 9 **Average AUC values for all methods regarding Table 3**. Table 3 was obtained based on these values. Click here for file

Additional file 10 **R-code for obtaining average AUC values for AFX data across six test sites when top *X *genes are defined as DEGs**. Two input files are required. One is the AFX gene expression data file obtained from http://edkb.fda.gov/MAQC/MainStudy/upload/norm_MAQC_AFX_123456_qPLIER16.zip. The other includes the DEG information derived from individual RT-PCR data. Specifically, the filename should be "data_TAQ.txt" (default) or "data_GEX.txt", generated by executing Additional file 2 or 3, respectively. After execution of this R-code, a fraction of the average AUC values for the AFX data when top ***X ***genes are defined as DEGs (***X ***= 100 by default), shown in Additional file 9, can be obtained in an output file arbitrarily named "result_AFX_100.txt". Note that the website with the normalized MAQC data was unavailable under review. The raw data may be available from http://www.fda.gov/ScienceResearch/BioinformaticsTools/Arraytrack/ or http://www.ncbi.nlm.nih.gov/geo/query/acc.cgi?acc=GSE5350. Click here for file

Additional file 11 **POG values for given numbers of top-ranked genes among three test sites**. (a) AFX data, (b) ABI data, (c) AG1 data, (d) GEH data, and (e) ILM data. Number of DEGs (***X ***) is shown on ***x ***-axis (log-scale). Percentage of genes (POG) for ***X ***top-ranked genes among three test sites is shown on ***y ***-axis. Click here for file

Additional file 12 **Raw data for Additional file **11. There are a total of ten sheets (five platforms × two comparisons). Click here for file

Additional file 13 **Correlation coefficients of ranked gene lists between any two test sites**. Click here for file

Additional file 14 **Correlation coefficients of 186 ranked lists of gene sets between any two test sites**. Click here for file

Additional file 15 **Raw data for Figure 2**. There are a total of ten sheets (five platforms × two comparisons). Click here for file

Additional file 16 **R-code for making left side of Figure 2(a)**. The R-code assumes the input filename is "POG_AFX_KEGG_AB.txt," which contains data for the first sheet (i.e., the sheet named "AFX_AvsB") in Additional file 15. Click here for file

Additional file 17 **Numbers of genes belonging to each of 186 KEGG Pathway gene sets in individual platforms**. Click here for file

Additional file 18 **Raw data for Figure 3**. There are two sheets. Click here for file
