# Supplementary figures and images for: Evaluating methods for ranking differentially expressed genes applied to microArray quality control data
Source: BMC Bioinformatics. 2011 Jun 6;12:227. doi: 10.1186/1471-2105-12-227 (PMC3128035; doi:10.1186/1471-2105-12-227)

## Slide 1
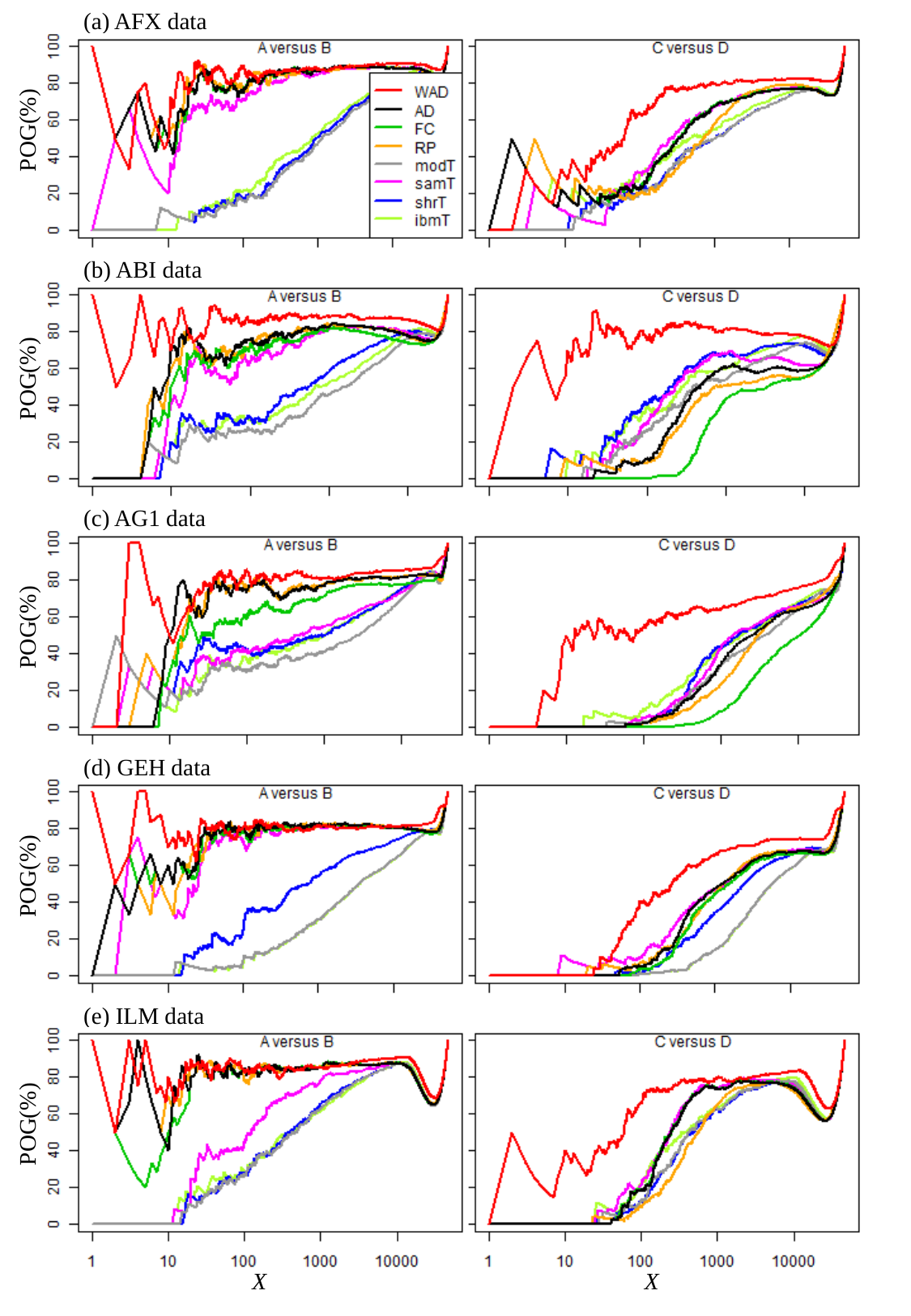

(a) AFX data
POG(%)
(b) ABI data
POG(%)
(c) AG1 data
POG(%)
(d) GEH data
POG(%)
(e) ILM data
POG(%)
X
X

Supplement: Additional file 11 — POG values for given numbers of top-ranked genes among three test sites. (a) AFX data, (b) ABI data, (c) AG1 data, (d) GEH data, and (e) ILM data. Number of DEGs (X ) is shown on x -axis (log-scale). Percentage of genes (POG) for X top-ranked genes among three test sites is shown on y -axis. [file 1471-2105-12-227-S11.PPT]
